# Neutron Depth Profiling: Overview and Description of NIST Facilities

**DOI:** 10.6028/jres.098.008

**Published:** 1993

**Authors:** R. G. Downing, G. P. Lamaze, J. K. Langland, S. T. Hwang

**Affiliations:** National Institute of Standards and Technology, Gaithersburg, MD 20899; Korea Research Institute for Standards and Science, Taedok Science Town, Taejon, Korea 305–606

**Keywords:** boron, cold neutrons, lithium, NDP, neutron depth profiling, nitrogen, oxygen, silicon, surface analysis

## Abstract

The Cold Neutron Depth Profiling (CNDP) instrument at the NIST Cold Neutron Research Facility (CNRF) is now operational. The neutron beam originates from a 16 L D_2_O ice cold source and passes through a filter of 135 mm of single crystal sapphire. The neutron energy spectrum may be described by a 65 K Maxwellian distribution. The sample chamber configuration allows for remote controlled scanning of 150 × 150 mm sample areas including the varying of both sample and detector angle. The improved sensitivity over the current thermal depth profiling instrument has permitted the first nondestructive measurements of ^17^O profiles. This paper describes the CNDP instrument, illustrates the neutron depth profiling (NDP) technique with examples, and gives a separate bibliography of NDP publications.

## 1. Introduction

The National Institute of Standards and Technology has operated since 1982 a dedicated NDP facility [1] using thermal neutrons at the NIST reactor. This paper describes applications of the NDP technique, presents a new cold neutron depth profiling (CNDP) instrument located on the CNRF at the NIST reactor, and gives in the Appendix an extensive bibliography of NDP publications as of July 1991.

In 1972 Ziegler et al. [2] first reported the development of a near-surface technique which has come to be known as neutron depth profiling (NDP). NDP is an isotope specific, nondestructive technique for the measurement of concentration versus depth distributions in the near-surface region of solids. This technique uses neutron induced reactions to measure the concentration versus depth profiles of a number of the light elements. NDP allows the first few micrometers of nearly any condensed material to be probed nondestructively. Biersack and coworkers [3,4] at the Institut Laue-Langevin facility in Grenoble subsequently advanced the technique to much of its present capabilities.

Since its introduction, over 100 articles have been published (see Appendix) describing the use of NDP to investigate materials and effects directly relating to materials research. The widespread application of NDP has been limited primarily by the number of intense neutron sources available—nuclear research reactors. Besides the NIST facilities, the United States has four other NDP facilities in use or under development: the University of Michigan Ford Nuclear Reactor [5,6], Texas A&M University [7], University of Texas at Austin [8], and North Carolina State University [9]. This activity, much of it recent, indicates that NDP has significant potential for materials research, and particularly for semiconductor research.

## 2. Fundamentals of the Technique

### 2.1 Physics

Lithium, beryllium, boron, sodium, and a number of other elements, have an isotope that, upon capturing a thermal neutron, undergoes an exoergic charged particle reaction. These reactions produce either a proton or an alpha particle, depending upon the isotope, and a recoiling nucleus. Each emitted particle has a specific kinetic energy defined by the *Q*-value of the reaction which in turn serves to identify the element. For the case of lithium, the reaction proceeds as

$${\;}^{6}\mathrm{Li}+n\to^{4}\mathrm{He}\left(2055\;\;\mathrm{keV}\right){+}^{3}H\left(2727\;\;\mathrm{keV}\right).$$ (1)

Four elements, Li, Be, B, and Na, are particularly well suited for the NDP technique since their neutron cross sections are quite large relative to other particle-producing reactions (see Table 1). In principle, there are essentially no interferences and profiling is permissible for all host materials. In practice, however, background contributions arise from energetic electrons and photons when analyzing materials that contain elements with significant (n, γ) cross sections.

To obtain a depth profile, a well-collimated beam of low energy neutrons (<10^−2^ eV) is used to illuminate a sample volume uniformly. While most of the neutrons pass through the sample without interacting, those sites containing reactive atoms capture neutrons in proportion to the capture cross section of the nuclide and act as an isotropic source of monoenergetic charged particles. The particles travel outward in essentially straight paths and lose energy primarily through numerous interactions with the electrons of the matrix. The difference between the well-known initial energy of the particle and its residual energy upon emerging from the surface of the sample is directly related to the depth of origin for the particles (i.e., the site of the parent atom). The target chamber is kept under vacuum so that no additional energy is lost from the emerging particle as it travels between the sample surface and the detector. Because the low-energy neutron carries very little momentum, the reaction center of mass is coincident with the site of the parent atom.

Sample damage due to temperature rise is minimal during the analysis. In the worst case, if an entire 10^9^ n/cm^2^ s beam is stopped by boron reactions in a sample, the temperature would reach a steady-state value of only 0.7 K greater than ambient. This calculation assumes there is no heat removal other than radiative. Such an extreme example would contain the equivalent of a few millimeters thickness of pure ^10^B. The amount of target nuclide consumed during a typical analysis is only a few tens-of-thousands of atoms. Some damage does occur due to knock-on of the outgoing charged particles with the matrix atoms. Here again the damage is small compared to nearly any other "nondestructive" analytical technique.

The depth corresponding to the determined energy loss for the emitted particle is determined by using the characteristic stopping power of the material, as compiled by Ziegler [10] and others [11] or by estimating the stopping power for compounds using Bragg's law [12] (i.e., the linear addition of the stopping powers of individual elemental constituents). The chemical or electrical state of the target atoms has an inconsequential effect on the measured profile in the NDP technique. Only the concentration of the major elements in the material is needed to establish the depth scale through the relationship of stopping power.

**Fig. 1:**
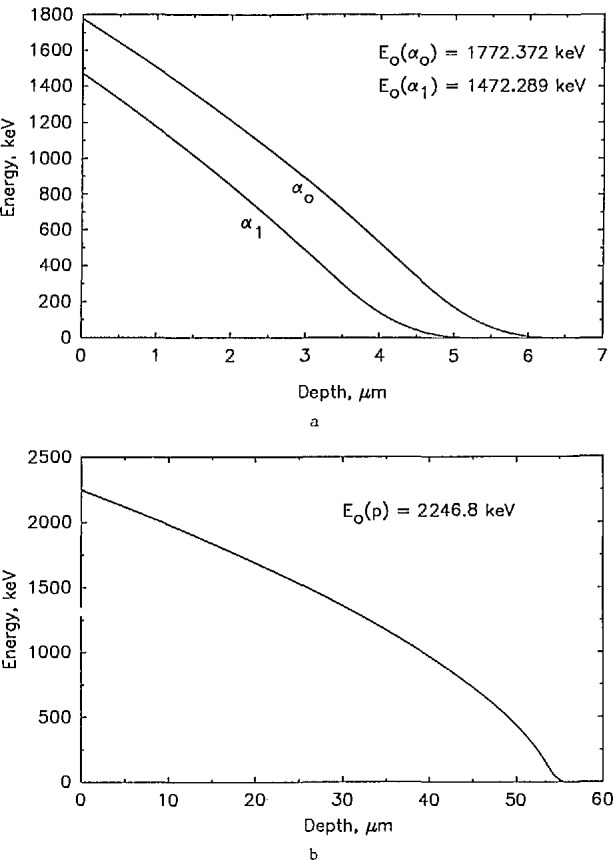
Plots depicting the relationship between the residual energy of charged particles, and depth of the originating nuclear reaction. Plot (a) gives the residual alpha particle energy versus depth for ^10^B in silicon, and plot (b) gives the relationship of the residual proton energy versus depth for ^22^Na in silicon.

Mathematically, the relationship between depth and residual energy can be expressed as

$$x=\int_{E(x)}^{E_{0}}dE/S\left(E\right),$$ (2)

where *x* is the path length traveled by the particle through the matrix material, *E*_0_ is the initial energy of the particle, *E*(*x*) is the energy of the emerging particle, and *S*(*E*) represents the stopping power of the material. Examples of the relationship between *x* and *E*(*x*) are displayed in Fig. 1 for ^10^B in silicon and ^22^Na in silicon.

### 2.2 Elemental Detection Limits

**Fig. 2:**
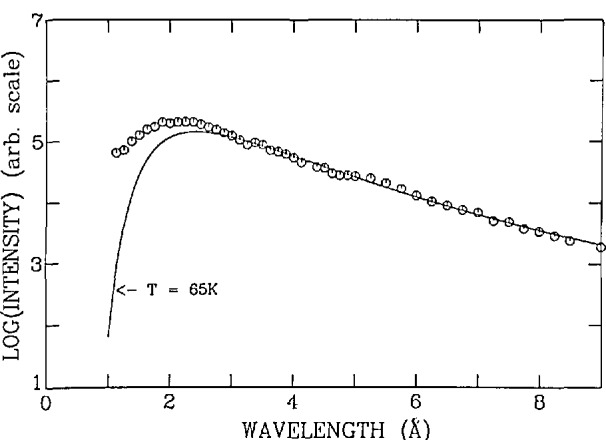
A eorreeted wavelength distribution, measured by time-of-flight, for the cold souree operating at 30 K with 7.5% H_2_O homogeneously mixed in the D_2_O. The solid curve is a Maxwellian speetrum for a temperature of 65 K that gave the best fit to the data from 3 to 9 Å.

The detection limit of the NDP method is directly proportional to the total neutron fluence and to the cross section of the reaction of interest. In the low-energy region, these cross sections are inversely proportional to the square root of the neutron energy. The lower the neutron energy, the greater the reaction rate. In a moderating medium, such as water, the neutrons, which start out with a few MeV of energy, are slowed down by successive collisions approaching temperature equilibrium with their surroundings. By lowering the temperature of the moderator, the average energy of the neutrons is also lowered (more commonly referred to as neutrons having a longer wavelength). Figure 2 gives the neutron distribution as a function of neutron wavelength for the NIST cold source; a 65 K Maxwellian distribution of neutrons is given for comparison. By integrating a reaction cross section over this distribution, one can obtain a spectrum-weighted average cross section for this neutron beam. The neutron beam is filtered with highest quality single-crystal sapphire so that epithermal neutrons and gamma radiation are preferentially scattered from the beam [13]. Although this filtering further reduces the cold source moderated neutron fluence rate by about 30 percent, there will be less radiation damage induced in sensitive materials such as polymers used in photoresists or ionic conductors. After taking into account the additional effect of the 135 mm of sapphire filter in the beam, the sensitivity of the CNDP instrument is increased by a factor of 1.7 solely from the effect of having lowered the energy of the neutrons from a thermal average distribution.

The number of charged particle counts collected in a data channel, of energy width d*E*, is directly proportional to the concentration of target atoms located within that corresponding depth interval. Upon calibrating the facility against an accurate isotopic standard, concentrations can be measured for that isotope (or other similar reactions) in subsequent samples, independent of the matrix, the concentration level, or location (within the depth that induced particles can escape the sample surface and be detected). Table 1 lists several properties for target atoms and the detection limits using the CNDP facility at the NIST reactor. Isotopes with charged particle cross sections of about a barn or greater are given. The conservative detection limits listed were calculated assuming 0.1 cps and a detector acceptance solid angle of 0.1 percent. Assuming a practical profiling depth of 2 *µ*m for the case of boron in silicon, boron concentrations down to the ppm (atom %) level can be accurately measured. The time required for an analysis is a function of the element and the desired accuracy. A boron implant of 1 × 10^15^ atoms per cm^2^ typically takes a few hours to obtain 1 percent precision (counting statistics) at most points along the profile curve. Since the background signal is almost negligible, a sample could be counted for tens of hours to obtain the required definition in the profile shape.

### 2.3 Reaction Product Energy Spectra

**Fig. 3:**
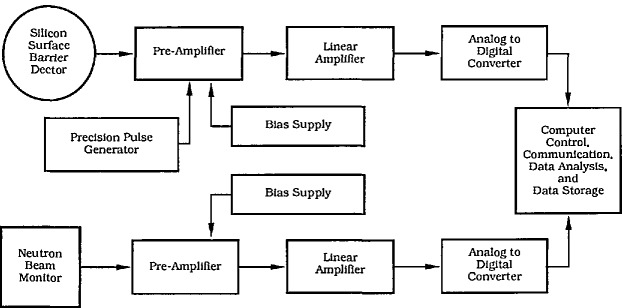
Basic diagram of the data acquisition, and analysis electronics for either NDP facility. Alterations to the upper half of the scheme are necessary for coincidence detection, and for time of flight detection systems.

The charged particle energy spectrum is collected using a transmission-type silicon surface barrier detector, electronic amplifiers, an analog-to-digital converter and a multichannel analyzer (see Fig. 3). For the NDP system at NIST, a reference pulse is also fed into the electronics to monitor the stability of the system thus allowing corrections to be made should electronic drift occur during the course of the measurement. Other NDP systems are described more specifically in the references [1,2,4,6,7,14–21]. By using a computer-based data acquisition system, the depth profile can be displayed in real time.

**Fig. 4:**
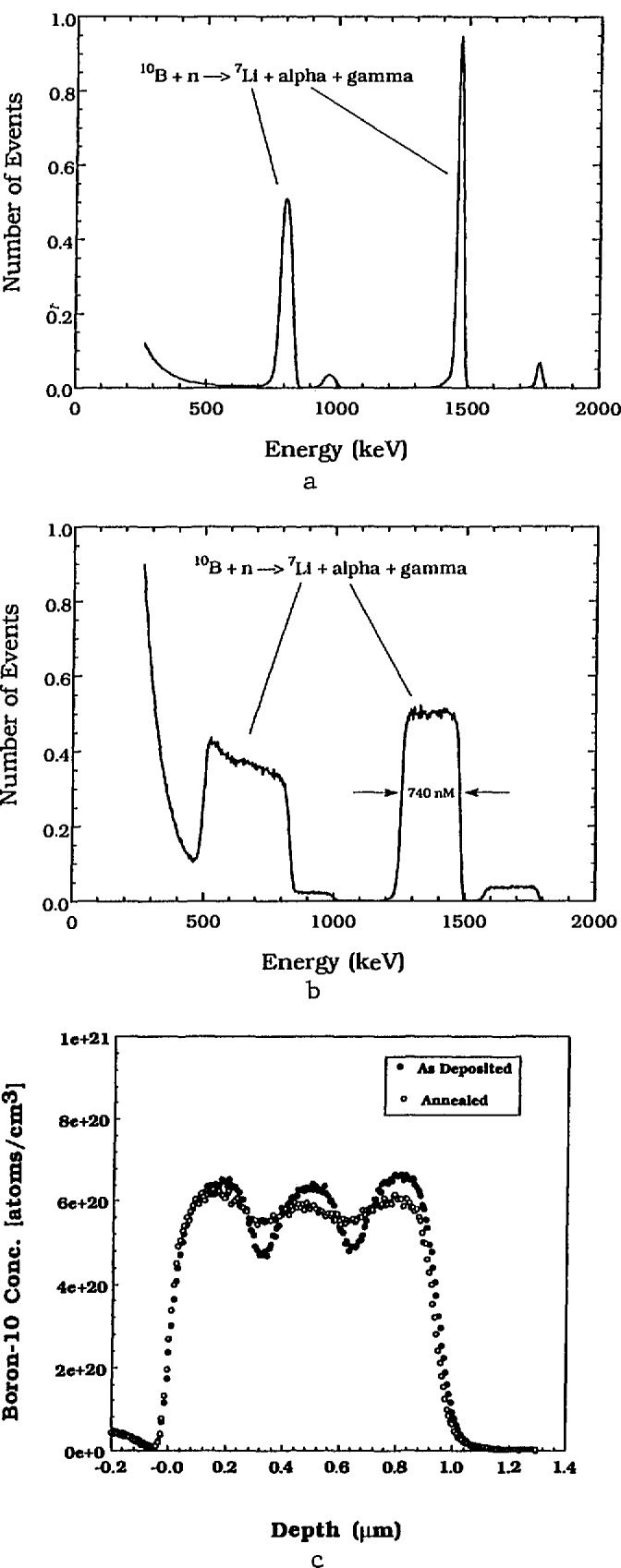
Energy profiles of particles emitted by the ^10^B reaction for (a) a 2 nm thick surface deposition, (b) a 740 nm thick borosilicate glass film on Si, and (c) the depth profile using only the 1472 kcV alpha particles from a borophosphosilicate glass film, 1.2 µm thick.

Examples of the detected energy spectra from three boron containing structures are shown in Fig. 4. With boron, 94 percent of the neutron reactions are

$$\begin{array}{l} {\;}^{10}B+n\to^{4}\mathrm{He}\left(1472\;\mathrm{keV}\right){+}^{7}\mathrm{Li}\left(840\;\;\mathrm{keV}\right)+\\ \gamma \left(478\;\;\mathrm{keV}\right) \end{array}$$ (3)

and 6 percent of the reactions [22] proceed as

$${\;}^{10}B+n\to^{4}\mathrm{He}\left(1776\;\mathrm{keV}\right){+}^{7}\mathrm{Li}\left(1013\;\;\mathrm{keV}\right).$$ (4)

Figure 4(a) is the energy spectrum of a 2 nm thick, surface deposit of boron on a nickel substrate. Figure 4(b) shows the energy distribution of particles from a 740 nm thick borosilicate glass (BSG) film on a silicon wafer substrate. Both figures show the fourfold redundancy [see Eqs. (3) and (4)] of depth profiles for a boron containing material. The 1472 keV alpha particle or its 840 keV ^7^Li recoil particle are typically used for the profile determinations because of their higher intensity, however, the remaining two peaks can serve to confirm the results. Figure 4(c) shows the depth spectrum of the 1472 keV alpha for a borophosphosilicate (BPSG) film with a periodic concentration variation from the surface down to the glass-silicon interface. The total thickness of this film is about 1.2 *µ*m.

### 2.4 Resolution

The broadening of the signal in Fig. 4(a) is primarily due to the energy resolution of the detector and associated electronics. In addition to the detector and system resolution, other factors that contribute to the depth resolution include: i) small-angle scattering of the charged particles within the sample, ii) energy straggling of the particles, and iii) the nonzero acceptance angle of the detector giving a spread in path lengths for particles from the same depth. These contributions to the resolution are treated by Biersack et al. [23] and by Maki et al. [24]. Item i) above can be seen as a low-energy tail on sharp spectral features appearing some three orders-of-magnitude less in intensity than the main feature.

Each material has a characteristic stopping power and, therefore, the resolution and the depth of profiling will vary in different materials. The lithium particle from the boron reaction has greater charge than its alpha counterpart and loses energy more rapidly allowing greater profile resolution; however, the alpha particles have the greater range and consequently allow deeper profiles to be obtained (typically 1 or 2 *µ*m). The full width at half maximum (FWHM) resolution in the depth profile obtained from the 1472 keV alpha of a boron reaction in silicon is typically a few tens of nanometers. On the other hand, protons from the ^22^Na(n,p)^22^Ne reaction give a resolution on the order of a few hundred nanometers, but can be used to profile 30 to 40 *µ*m in depth. Since for thermal or cold neutrons particle emission is isotropic, the detector can be placed at an angle with respect to the normal of the sample surface to view longer particle path lengths from the same sample depth. The depth resolution is improved in this fashion and has been shown to be as well defined as 7 nm (FWHM) for the case of boron in silicon [23,25]. Small concentration variations in the first nanometer of a sample surface can often be identified by comparing differentiated spectra of known homogeneous standards with that of differentiated spectra of unknown samples. Deconvolution algorithms used to unfold the system response function from collected energy spectra [2,24,26–30] have provided improvement in depth resolution by greatly reducing system resolution broadening. With some *a priori* knowledge of the sample, modeling of the spectrum reveals subtle concentration variations.

Improvements to detection limits for NDP require either more intense neutron sources or changes in basic instrumental design. Using larger detectors for greater solid angles are a more efficient use of the existing neutron fluences, however, the energy resolution is degraded. As the energy resolution of charged particle detectors improves there is a corresponding gain in profile resolution. Better algorithms for the deconvolution of system response from the energy spectrum are necessary as well. Fink et al. [16] have described a charged particle energy analyzer for NDP using electromagnetic focusing that should improve the energy resolution, while reducing the photon induced background levels.

Another approach is the use of a coincidence technique [31]. If the sample is thin enough to allow both the light particle and the recoil nucleus to escape from opposing surfaces of the sample, two detectors can be used to detect both particles simultaneously. By requiring a coincidence between the two detectors, background interferences are reduced [32] and the solid angle of collection can be increased by 10 to 100 fold by bringing the detector closer to the sample. The depth resolution is improved because there is no dependency upon solid angle of acceptance to the detector. Mathematically this is made possible by the fact that the sum of the energy loss and the residual energy of the two reaction products must equal the *Q*-value of the reaction. The major disadvantages are that the sample must be thin enough to permit the escape of both reaction products and that only a few elements are applicable to this method.

Both neutron intensity and gain in spatial resolution will be possible with a neutron focusing device currently being developed at NIST [33]. Long wavelength neutrons are guided to areas of a few mm square providing locally high neutron fluences. This will permit the development of two- and three-dimensional neutron depth profiling. The use of position sensitive detectors and ion optics can further accelerate progress toward three dimensional nondestructive depth profiling.

## 3. Applications

The development of the neutron depth profiling technique has been motivated by the importance of light elements in optical, polymer, metal alloy and especially microelectronic materials. Boron is widely used as a *p*-type dopant in semiconductor device fabrication and in the insulating passivation barriers applied either as an organometallic or a vapor phase deposition of borosilicate glass. NDP has both good sensitivity for boron and good spatial resolution to a depth of a few micrometers. It is used both as a stand alone technique and in a complementary role with a variety of other analytical methods [34–37]. Recently NDP has been used to certify the concentration and confirm the profile of boron in silicon for a NIST Standard Reference Material (SRM 2137) primarily for the use of secondary ion mass spectroscopy (SIMS) calibration.

Applications of NDP are quite diverse as can be seen by the titles of the articles in the appendix. Although an exhaustive discussion of all the uses would be beyond the scope of this paper, a few examples are given to illustrate its strengths.

### 3.1 Implantation

Ziegler and coworkers [2,18,38,39] introduced NDP by determining the range and shape of boron implantation distributions in doped and intrinsic silicon wafers. With the resultant profiles, they were able to calculate diffusion coefficients for boron in crystalline, amorphous, and arsenic doped silicon. Since little experimental data existed for the case of boron to judge the validity of the current range theories, the shape of the boron profiles from NDP were of great interest. NDP and other techniques have since been able to show that a Pearson IV model rather than a Gaussian profile describes more accurately the implant distribution [24,28,40–42].

In subsequent experiments, Biersack et al. [43] used the boron (n, *α*) reaction to show the effect of pre- and post-irradiation damage on boron implantation profiles. By post-irradiating a boron implant in silicon with 200 keV H^2+^, a migration of the boron to the induced damage sites was observed. In the same paper, diffusion and trapping of lithium ions in niobium were reported. Using the lithium (n, *α*) reaction, irradiation induced crystal defects were mapped through a depth of several micrometers for a variety of sample treatment conditions.

Of significant interest is the fact that determinations by NDP induce negligible damage to most materials. Sample surfaces are neither sputtered, as observed with SIMS, nor is the sample matrix altered. The thermal neutrons carry an insignificant amount of momentum into the material and induced reactions are of such low intensity that radiation damage is usually negligible. This allows precisely the same sample volume to be subjected to different processing conditions and to be examined by NDP at each stage. The sample may thereafter be passed to another analytical method such as SIMS, Rutherford Back Scattering (RBS), Proton Induced X-ray Emission (PIXE), Spreading Resistence Profiling (SRP), or Atomic Emission Spectrometry (AES) to obtain complementary data on the material. Analysis of the same sample by different methods allows extensive experimental testing of possible variability between samples or even across a single sample. As a result, NDP has been used as a reference technique for other methods of analysis [34]. If radioactive nuclides are formed during an analysis, it may not be desirable to place the sample in a sputtering-type instrument, thus avoiding possible contamination of sensitive detectors. This is certainly not the case for silicon wafers and most other electronic materials due to their very small neutron activation cross sections.

**Fig. 5:**
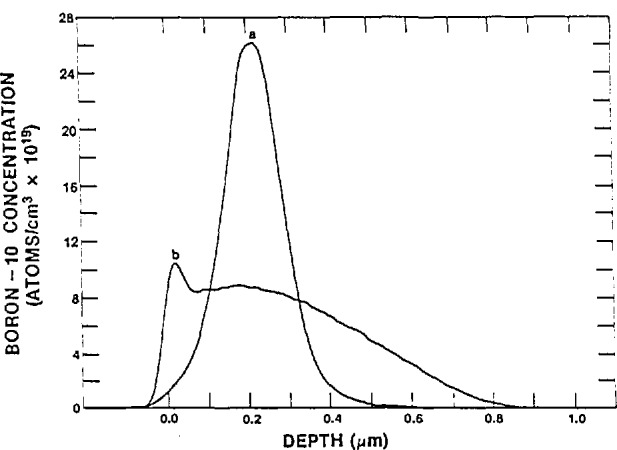
NDP depth profiles for a 70 keV ^10^B implant in silicon at a dose of 4 × 10^15^ atoms per em^2^. Depicted are (a) the as-implanted profile, and (b) after a 30 min anneal at 1000 °C, and indicating air leak during anneal.

Some of the features observed for an NDP profile are illustrated in Fig. 5. Curve 5(a) is an NDP profile for a 70 keV ^10^B implant in silicon at a total dose of 4 × 10^15^ atoms per cm^2^. To prevent channelling of the boron, the implant was made nominally 7° off normal in silicon cut perpendicular to the < 111 > surface. Curve 5(b) is the same wafer after being annealed at 1000 °C for 30 min. The diffusion broadening bounded by the surface is clearly apparent. The apparent boron concentration above the surface is an artifact of the detector resolution. Of particular interest is the small peak near the surface. A small unintentional air leak into the nitrogen back-filled annealing furnace allowed a thin film of SiO_2_ to grow on the silicon wafer surface. The segregation coefficient of boron between Si and SiO_2_ favors movement of the boron in the direction of the SiO_2_. Boron, as a consequence, was extracted from the bulk Si wafer into the surface SiO_2_. In a similar case Downing et al. [44] have shown that the native oxide (1.0–1.5 nm) that appears on nearly all Si surfaces is contaminated with boron at a level of 10^12^–10^13^ atoms/cm^2^.

Boron profiles by NDP in mercury cadmium telluride, an infrared detector material, have been measured by Ryssel et al. [45], Vodopyanov et al. [46], and Bowman [47]. Cervena et al. [30] have used NDP to study the implantation profiles of ^10^B in several photoresists used in masking operations and to determine range values for implants in several types of grown or deposited SiO_2_ films.

### 3.2 Interfacial Profiling

Neutron depth profiling is well suited for measurements across interfacial boundaries. Kvitek et al. [27] and others [20,21,28,34] have studied profiles of boron implanted and diffused across the interfacial region of Si/SiO_2_. Other NDP experiments [48,49] have been described for interfaces of silicon, silicon dioxide or metal on metal, where diffusion distributions and segregation coefficients were studied.

**Fig. 6:**
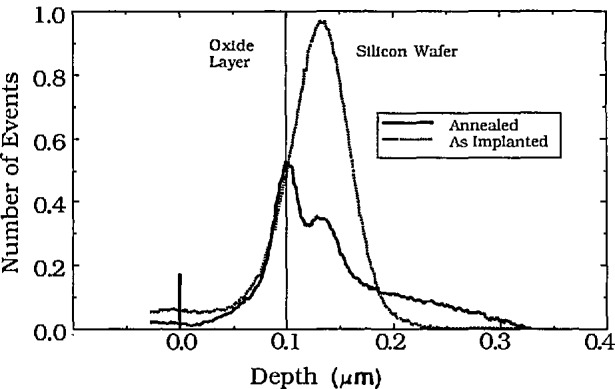
NDP depth profiles for a 70 keV ^10^B implant to a dose of 1 × 10^16^ in an Si wafer that had a 0.2 *µ*m film of thermally grown SiO_2_ (a) as deposited, and (b) after a 30 min anneal at 1000°C.

Knowledge of stopping powers for the major elemental constituents is the primary requirement to establish the depth scale. Figure 6 depicts an NDP profile of boron across an SiO_2_ — Si interface. Boron was implanted to a dose of 1 × 10^16^ atoms per cm^2^ at 70 keV into a silicon wafer that had 0.2 *µ*m of thermally grown SiO_2_ covering the surface. The ^7^Li particle energy spectrum from the ^10^B(n,*α*)^7^Li reaction was used for this profile to increase the depth resolution. Notice the smooth transition of the as-implanted boron concentration across the interfacial region. Although the FWHM depth resolution is on the order of 10–15 nm, it is clear that no discontinuity exists at the interface of the two materials. The same region is shown again after annealing the sample for 30 min at 1000 °C [34]. At the mean depth of the original implant, a residual peak remains. The solid solubility of boron in silicon had been exceeded in the original implant which is suspected [50] to give rise to Si-B compounds. Since the diffusivity of boron is much less in silicon dioxide than in silicon, the boron on the silicon side of interface migrates into the bulk silicon while the boron on the SiO_2_ side of the interface remains essentially immobile during the annealing. The segregation coefficient of boron between Si and SiO_2_ favors the SiO_2_ which accounts for the increase in boron concentration at the interface analogous to the effect seen in Fig. 5(b).

Matsumura et al. [19,51] discussed the use of the NDP method to investigate the diffusivity of boron in hydrogenated amorphous silicon (a–Si:H), an important material in solar cell production. When using a *p*-type/intrinsic/*n*-type (*p-i-n*), layered amorphous silicon structure, the boron from the 60 nm thick *p*-type layer was observed to diffuse into the underlying undoped a–Si:H layer. From these measurements, they were able to calculate the activation energy and diffusion coefficient for boron in a–Si:H (the latter being a dramatic 12 orders of magnitude larger than for crystalline silicon) and estimate the deterioration rate of boron-doped solar cells.

### 3.3 Channel Blocking

Arrayed-charged particle detectors [40] are used with the NDP technique to determine both the energy and lateral position of emitted particles. Similar to RBS performing channel blocking experiments, NDP is used to discern between interstitially or lattice located atoms, but only those isotopes which are charged particle emitters. The minor damage incurred from thermal neutron induced reactions is negligible when compared to RBS which bombards the sample with highly energetic charged particles. It therefore seems appropriate that one of the first applications of NDP was to establish the depth and lattice position of dopants in single crystal materials [15,52].

Using NDP, Fink et al. [16] have reported variations in the lattice position of the dopant atoms with respect to the depth and temperature treatment for boron implants in silicon. One example, where a boron implant of 1 × 10^16^ atoms per cm^2^ was made at 120 keV and annealed at 1000 °C for 1 h, showed that two thirds of the boron atoms located near the average range of the implant remained unordered. The remaining one third in that region were shown to be interstitial. The further from the average range of the implanted atoms, both above and below the plane, the more nearly substitutional the boron atoms were in the matrix. The largest component of the total boron implanted in these regions, however, remained randomly located in the lattice.

In the past, researchers [53] have used etchable acetate foils to map the channel blocking pattern, analogous to the nuclear track technique (NTT) method of particle counting. However, quantitative analysis becomes tedious with this method and little depth information is obtained. A review of channel blocking by NDP for boron in silicon is presented by Fink et al. [16]

### 3.4 Thin Films and Leaching

Materials for optical waveguides and fiber optics depend on uniform composition to prevent changes in the refractive index of the material, which can reduce the intensity of signal transmissions. Similar materials are used in thin, insulating overcoats on electronic devices. The high solubility and mobility of boron and lithium in these technologically important materials make them susceptible to leaching during wet processing, annealing at elevated temperatures, and during the cutting or polishing of surfaces. Riley et al. [54] have studied some of the effects that the processing steps can have on boron in the near surface region of fiber-opticgrade glasses. Using NDP, SIMS, Nuclear Track Technique (NTT), and prompt gamma activation analysis (PGAA) to quantify and map the boron distribution, they were able to show that a significant amount of leaching occurs within the first few micrometers of the samples when a fine grinding cut was made in the presence of an aqueous coolant. The leaching of boron from the near surface is obvious and can be attributed to the action of the water during the cutting step. In their study, Riley et al. demonstrated that leaching could be avoided by substituting a glycol based liquid for the water coolant during the cut.

For a sufficiently thin film, such as a BSG overcoat on a silicon wafer, a single NDP spectrum is capable of revealing the thickness, the boron distribution profile, and the total amount of boron present. Changes in the film can be quantified subsequent to wafer annealing. This processing is designed to drive out trapped reaction products in the CVD process and remove trapped voids from the glass film. Also, the effect of reflowing the glass film on the original boron profile can be shown, including boron loss and diffusion into the substrate [55].

## 4. The CNDP Instrument

**Fig. 7:**
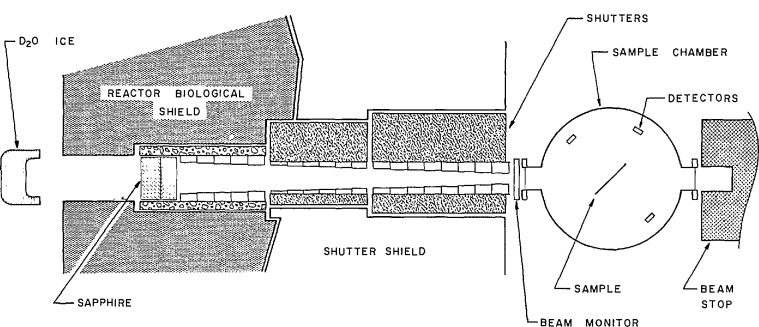
A schematic layout of Cold Tube-West showing the relative positions of the cold source, sapphire filters, collimators, and the sample chamber.

The cold neutron source in the NIST research reactor (called NBSR) is a block of D_2_O−H_2_O (7.5%–H20) ice cooled to ~45 K by recirculating helium gas. The gas is circulated by a compressor through a refrigerator capable of removing 1 kW of heat at 20 K. A lead-bismuth shield removes most of the gamma heating before it reaches the cryostat and cold moderator. Figure 7 indicates the layout of Cold Tube West (CT-W) on which the NDP instrument is located. The neutrons are filtered by 135 mm of single crystal sapphire which has the effect of reducing the slow neutron fluence rate by 1/3, but the fast neutron fluence rate by a factor of about 500. Collimators are located both within the biological shield and in the external-to-the-shield rotating shutter. The shutter is two cylinders, whose beam tubes fully align in the beam open configuration and are nonaligned in the beam closed configuration. The collimator pieces in these shutters can be accessed in the beam closed configuration with the reactor at full power. The diameter and intensity of the neutron beam can then be modified at any time to suit the needs of a particular experiment. The measured neutron fluence rate (capture flux) at the target position with the 16 mm diameter collimator is 1.2 × l0^9^ cm^−2^ s^−1^. A pancake fission chamber mounted on the entrance port of the NDP chamber provides a run to run monitor.

The target chamber was obtained from a commercial vendor using a design developed at NIST. The entire chamber is stainless steel and uses copper gaskets at all but three sealing surfaces: the beam entrance and exit windows and the opening used for changing samples. This last surface can use either viton O-rings or copper gaskets. In practice, the desire to change samples quickly usually outweighs the need for ultra high vacuum, but that capability is readily accessible. The chamber itself is a 610 mm diameter cylinder with access ports in the top and bottom plates as well as through the side walls. All flanges conform to standard Confiat^1^ flange specifications making it possible to add new features to the chamber. Some of these include *in situ* cleaning of samples, time-of-flight measurements, heating and cooling of the sample, and cooling of the surface barrier detectors.

The beam enters and exits through 100 mm diameter ports sealed with thin aluminum windows. These can be replaced with sapphire windows if a metal gasket seal is required. The chamber is evacuated with a 180 L/s magnetic bearing turbo molecular pump. This pump was chosen to reduce microphonic effects on the charged particle detectors. The detectors are transmission-type surface barrier detectors in a ring mount. A rotary base positions the charged particle detectors about the axis of the sample. Detectors can be placed at any angle and detectors can be mounted every 10°. Currently, there exists electronics to operate four detectors simultaneously.

**Fig. 8:**
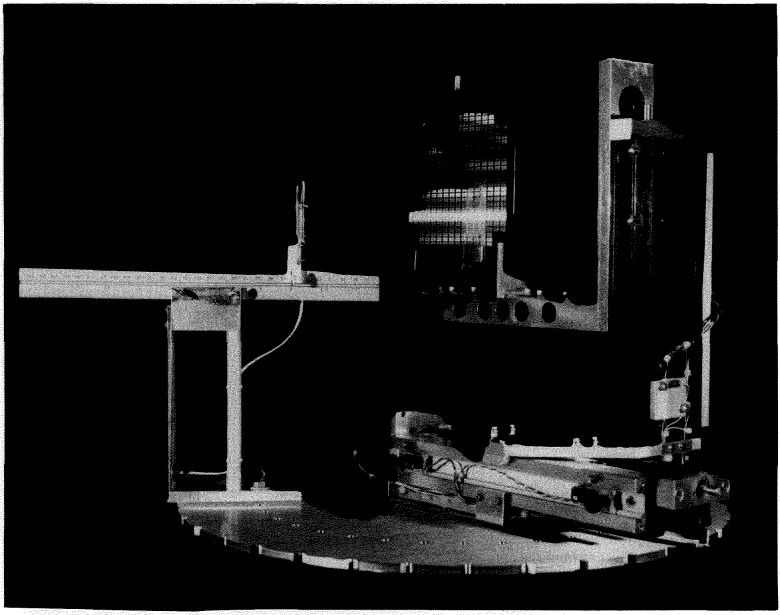
A photograph of the CNDP sample holder/positioning equipment without the vacuum chamber. A 150 mm silicon wafer is shown in sample position for a sense of dimensions. The sample can be independently translated in two-dimensions and rotated. A transmission-type surface barrier detector is on the right which is mounted on an independently rotatable base.

Samples (up to 200 mm in diameter) are mounted on a set of motor driven positioners. A second rotary base selects the angle of the sample with respect to the beam. The ability to rotate the detectors and sample independently allows the detector to be positioned at any angle with respect to the sample without putting the detector in the beam. Mounted on top of the sample rotator are *x* and *y* positioners. These have 150 mm of travel each, allowing a full scan of 150 × 150 mm sample areas. All four positioning devices are controlled by a PC compatible microcomputer. Figure 8 is a photograph of the interior of the CNDP target chamber. A program has been written in BASIC to enable unattended sample scans. The signals from the detectors are processed in a standard fashion and are interfaced with a multiuser minicomputer. This computer can simultaneously process data from both the thermal and cold NDP facilities. Spectra from these computers can then be transferred to a variety of other computers for data reduction, plotting, etc. A comparison of several characteristics of the two NDP facilities at NIST is given in Table 2.

**Fig. 9:**
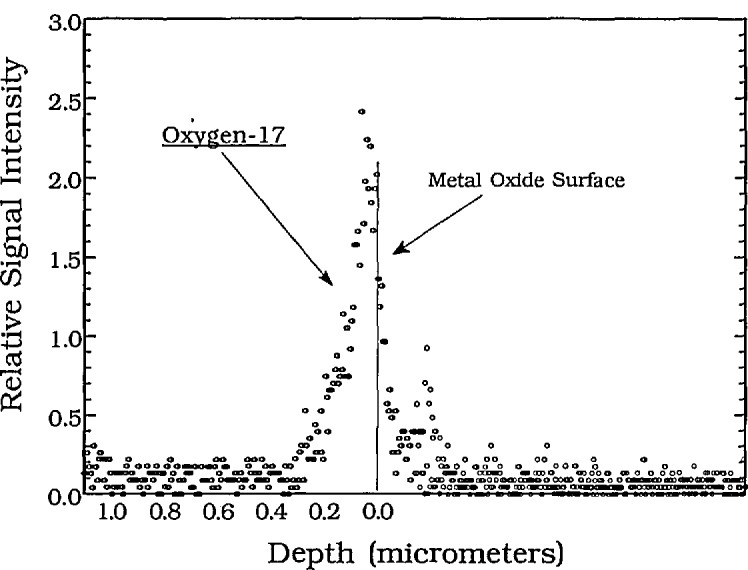
The profile of oxygen-17 obtained using the cold NDP instrument. The sample was Cobalt-Nickel Oxide enriched with ^17^O. The small boron peak is due to a contaminant on the surface of the sample. See text for description.

Figure 9 is an ^17^O(n,*α*)^14^C profile taken at the CNDP facility. The sample of Cobalt Nickel Oxide (enriched to 50% ^l7^O) was prepared by Eastman Kodak. A surface boron contamination is observed to the right of the oxygen profile. Because the unattenuated energy of the alpha from the boron reaction is higher in energy (1472 keV) than that of the full energy alpha from the oxygen (1413 keV), the boron profile appears as an artifact peak “above” the surface on the oxygen depth scale. Adjustment of the depth scale will produce a boron concentration profile. To our knowledge, this is the first nondestructive determination of near surface oxygen made by NDP.

## 5. New Capabilities

Several features are planned for the CNDP instrument in addition to those discussed in the above section on detection limits [22]. This includes cooling of the charged particle detectors which has the effect of reducing the thermal induced electronic noise present in the detector. Ten or more percent improvement in the detector resolution is to be expected by cooling the detector to liquid nitrogen temperatures. Another planned feature of the CNDP system is sputter cleaning by low energy ion beams of sample surfaces in the target chamber. This is important for ultra high vacuum applications, particularly when a time of flight detector is being used. For both UHV and normal applications sputtering can be employed to remove surface layers systematically for profiling deeper into the material. *In situ* heating and cooling of samples for diffusion and annealing studies will also enhance the usefulness of the NDP facility.

## 6. Summary

NDP provides an isotope specific, nondestructive technique for the measurement of concentration versus depth distributions in the near-surface region of solids. The simplicity of the method and the interpretation of data have been described. Major points to be made for NDP as an analytical technique include: i) it is nondestructive; ii) isotopic concentrations are determined quantitatively; iii) profiling measurements can be performed in essentially all solid materials with depth resolution and depth of analysis being material dependent; iv) it is capable of profiling across interfacial boundaries; and v) there are few interferences. The profiles are generated in real-time, analyzing depths of up to tens of micrometers. NDP is applied to many areas of materials research, as discussed here and in the references given in the Appendix. With the installation of the CNDP facility the ability to obtain oxygen profiles as well as those for chlorine or sulfur is now possible adding to the elements previously analyzed at NIST: boron, lithium, nitrogen, sodium, beryllium, and helium.

## Figures and Tables

**Table 1:** Summary of the NDP reaction characteristics and example detection sensitivities for the 20 MW NIST reactor

Elem.	Reaction	% Abundance or (atoms/mCi)^a^	Energy of emitted particles (keV)		Cross section (barns)	Detection limit (atoms/cm^2^)^b^
He	^3^He(n,p)^3^H	0.00014	572	191	5333	1.5 × 10^12^
Li	^6^Li(n,*α*)^3^H	7.5	2055	2727	940	9.0 × 10^12^
Be^a^	^7^Be(n,p)^7^Li	(2.5 × 10^14^)	1438	207	48000	1.7 × 10^11^
B	^10^B(n,*α*)^7^Li	19.9	1472	840	3837	2.1 × 10^12^
N	^14^N(n,p)^14^C	99.6	584	42	1.83	4.5 × 10^15^
O	^17^0(n,*α*)^14^C	0.038	1413	404	0.24	3.5 × 10^16^
Na^a^	^22^Na(n,p)^22^Ne	(4.4 × 10^15^)	2247	103	31000	2.3 × 10^11^
S	^33^S(n,*α*)^30^Si	0.75	3081	411	0.19	6.0 × 10^16^
Cl	^35^Cl(n,p)^35^S	75.8	598	17	0.49	1.7 × 10^16^
K	^40^K(n,p)^40^Ar	0.012	2231	56	4.4	1.9 × 10^15^
Ni^a^	^59^Ni(n,*α*)^56^Fe	(1.3 × 10^20^)	4757	340	12.3	7.0 × 10^14^

a Radioactive species.

b Dctection limit based on 0.1 cps, 0.013 Sr detector solid angle, and a neutron intensity of 6 × 10^9^ s^−1^.

**Table 2:** Comparison of the two NIST facilities used for NDP. The thermal NDP facility is located at beam tube 3 (BT-3), and has been operational since 1982. The cold NDP facility is a CNRF instrument located at cold tube-west (CT-W), and has been operational since November 1990

	BT-3 (thermal)	CT-W (cold)
Thermal equivalent fluence rate	4×10^8^ cm^−2^ s^−1^	1.2 × 10^9^ cm^−2^ s^−1^
Sapphire filtering	200 mm	135 mm
Peak neutron energy	22.5 meV	≈8 meV
Relative sensitivity	1	3
Gamma dose	400 mR/h	≈400 mR/h
Maximum sample size	100 × 100 mm	200 × 200 mm
Typical beam diameter at sample position	13 mm	30 mm
Number of detectors	2	4 (more possible)
Remote sample, and detector rotation	Yes	Yes
Incremental rotational detector movement	0.001°	0.025°
Sample scanning	No	Yes
Incremental rotational sample movement	0.01°	0.025°
Incremental translational sample movement (*X−Y*)	Hand positioned	3.2 *µ*m
UHV capability	No	Yes

## Footnotes

1 Certain commercial equipment, instruments, or materials arc identified in this paper to specify adequately the experimental procedure. Such identification does not imply recommendation or endorsement by the National Institute of Standards and Technology, nor does it imply that the materials or equipment identified arc necessarily the best available for the purpose.

## 8. Appendix A Neutron Depth Profiling Bibliography

Alfassi, Z. B., and Yang, M. H. (1990). Depth Profiling of Silicon by Nuclear Activation Methods. In Activation Analysis, Z. B. Alfassi, ed., Boca Raton, FL, CRC Press, pp. 579–606.
Bancrjee, I., Frost, M. R., Davies, P. W., Cox, J. N., and Downing, R. G. (1989). SIMS, and Neutron Depth Profiling Studies of SiO_2_/Si_3_N^4^/SiO_2_/Si Structures. In Secondary Ion Mass Spectrometry (SIMS VII), A. Benninghoven, C. A. Evans, K. D. McKeegan, H. A. Storms, and H. W. Werner, eds., Monterey, CA, John Wiley, and Sons, pp. 235–238.
Biersack, J. P. (1983). He Profiles in Various Metals after Implantation, and Thermal Anneals. Radiation Effects **78**, 363.
Biersack, J. P., and Fink, D. (1973). Observation of the Blocking Effeet after ^6^Li(n,t)'^l^He Reactions with Thermal Neutrons. Nuclear Instruments and Methods **108**, 397–399.
Biersack, J. P., and Fink, D. (1974). Damage, and Range Profiles of Lithium Implanted into Niobium. Journal of Nuclear Materials **53**, 328–331.
Biersack, J. P., and Fink, D. (1975). Channeling, Blocking, and Range Measurements Using Thermal Neutron Induced Reactions. In Atomic Collisions in Solids, S. Datz, B. R. Appleton, and C. D. Moak, eds., New York, Plenum Press, pp. 737–747.
Biersack, J. P., and Fink, D. (1975). Implantation of Boron, and Lithium in Semiconductors, and Metals. In Ion Implantation in Semiconductors, S. Namba, ed., New York, NY, Plenum Press, pp. 211–218.
Biersack, J. P., and Fink, D. (1975). Study of He Distributions in Niobium by Means of (n,p) Reactions. In International Conference on Radiation Effects, and Tritium Technology for Fusion Reactors, CONF-750989, Gatlinburg, TN, USERDA, pp. II362–II371.
Biersack, J. P., Fink, D., Henkelmann, R., and Müller, K. (1978). The Use of Neutron Indueed Reactions for Light Element Profiling, and Lattice Localization. Nuclear Instruments and Methods **149**, 93–97.
Biersack, J. P., Fink, D., Henkelmann, R. A., and Müller, K. (1979). Range Profiles, and Thermal Release of Helium Implanted into Various Metals. Journal of Nuclear Materials **85–86**, 1165–1171.
Biersack, J. P., Fink, D., Lauch, J., Henkelmann, R., and Müller, K. (1981). An Instrument for Lattice Location Studies of Light Impurity Atoms by Means of (n, *α*)-Rcactions. Nuclear Instruments and Methods **188**, 411–419.
Biersack, J. P., Fink, D., Mertcns, P., Henkelmann, R. A., and Müller, K. (1976). Helium Profiles in Niobium, and Molybdenum. In Plasma Wall Interactions, Oxford, Pergamon Press, pp. 421–430.
Bicrsack, J. P., Fink, D., Miekeley, W., and Tjan, K. (1986). 1–3 MeV Alpha, and Trition Stopping Powers in LiF, and Li Alloys. Nuclear Instruments and Methods **B15**, 96–100.
Bogáncs, J., Gyulai, J., Hagy, A., Nazarov, V. M., Seres, Z., and Szabó, A. (1979). Use of the Reaction ^10^B(n, *α*)^7^Li to Determine the Distribution of Boron Implanted in Silicon. Joint Institute for Nuelear Research **1**, 59–64.
Bogáncs, J., Szabó, A., Nagy, A. Z., Csokc, A., Pecznik, J., and Krakkai, I. (1979). Nondestructive Nuclear Method for Boron Analysis in Plant Samples. Radiochemical and Radioanalytical Letters 39(6), 393–403.
Bowman, R. C., Jr., Downing, R. G., and Rnudsen, J. F. (1987). NDP Evaluations of Boron Implanted Compound Semiconductors. In American Nuclear Society —Material Characterization Using Neutron Depth Profiling **55**, Los Angeles, CA, American Nuclear Society, pp. 212–214.
Bowman, R. C., Rnudsen, J. F., and Downing, R. G. (1990). Neutron Depth Profiling of Boron Implanted Semiconductors. In Materials Research Society **166**, North Holland, pp. 331–336.
Bowman, R. C., Jr., Rnudsen, J. F., Downing, R. G., and Rremer, R. E. (1988). Distribution of Boron Atoms in Ion Implanted Compound Semiconductors. In Materials Research Society **126**, pp. 89–92.
Bowman, R. C., Marks, J., Downing, R. G., Rnudsen, J. F., and To, G. A. (1987). Effects of Boron Implantation on Silicon Dioxide Passivated HgCdTe. In Materials Research Society **90**, pp. 279–286.
Bowman, R. C., Jr., Robertson, R. E., Knudsen, J. F., and Downing, R. G. (1986). Studies of Boron Implantations through Photochemically Deposited SiO_2_ Films on Hgl-xCdxTe. In Infrared Detectors, Sensors, and Focal Plane Arrays **686**, San Diego, CA, Society of Photo-Optical Instrumentation Engineers, pp. 18–25.
Cervená, J., Hnatowicz, V., Hoffmann, J., Kosina, Z., Rvítek, J., and Onheiser, P. (1981). The Use of the Induced Reaction for Boron Profiling in Si. Nuclear Instruments and Methods **188**, 185–189.
Cervená, J., Hnatowicz, V., Hoffmann, J., Kvítek, J., Onheiser, P., and Rybka, V., A. (1981). A Study of Masking properties of SiO_2_, and Photoresists with Boron Ion Implantation. Tesla Electronics **14**(1), 16–20.
Chu, W. R. (1989). Large Angle Coincidence Spectrometry for Neutron Depth Profiling. Radiation Effects and Defects in Solids **108**, 125–126.
Chu, W. R., and Wu, D. T. (1988). Scattering Recoil Coincidence Spectrometry. Nuclear Instruments and Methods **B35**, 518–521.
Coakley, R. J. (1991). A Cross-Validation Procedure for Stopping the EM Algorithm, and Deconvolution of Neutron Depth Profiling Spectra. IEEE Transactions on Nuclear Science 38(1), 9–15.
Cox, J. N., Hsu, R., McGregor, P. J., and Downing, R. G. (1987). NDP, and FTIR Studies of Borophosphosilicate CVD Thin-Film Glasses. In American Nuclear Society —Material Characterization Using Neutron Depth Profiling **55**, I. O. Macke, ed., Los Angeles, CA, American Nuclear Society, pp. 207–209.
Crowder, B. L., Ziegler, J. F., and Cole, G. W. (1973). The Influence of the Amorphous Phase on Boron Atom Distributions in Ion Implanted Silicon. In Ion Implantation in Semiconductors, and Other Materials, B. L. Crowder, ed., New York, Plenum Press, pp. 257–266.
Crowder, B. L., Ziegler, J. F., Morehead, F. F., and Cole, G. W. (1973). The Application of Ion Implantation to the study of Diffusion of Boron in Silicon. In Ion Implantation in Semiconductors, and Other Materials, B. L. Crowder, eds., New York, Plenum Press, pp. 267–274.
Deruytter, A. J., and Pelfer, P. (1967). Precise Determination of the Branching Ratio, and Q-Value of the ^10^B(n,*α*)^7^Li Reaction, and of the ^6^Li(n,*α*)^3^H Reaction. Journal of Nuclear Energy **21**, 833–845.
Downing, R. G. (1988). Neutron Depth Profiling: Current Developments of the Technique in the United States. In Industrial Radiation, and Radioisotope Measurement Applications **56**(3), I. O. Macke, ed., Pinehurst, NC, American Nuclear Society, pp. 15–16.
Downing, R. G., Fleming, R. F., Langland, J. R., and Vincent, D. H. (1983). Neutron Depth Profiling at the National Bureau of Standards. Nuclear Instruments and Methods **218**, 47–51.
Downing, R. G., Fleming, R. F., Maki, J. T., Simons, D. S., and Stallard, B. R. (1984). Near-Surface, and Interfacial Profiling by Neutron Depth Profiling (NDP), and Secondary Ion Mass Spectrometry (SIMS). In Thin Films, and Interfaces II, J. E. E. Baglin, D. R. Campbell, and W. R. Chu, eds., New York, North-Holland, pp. 655–656.
Downing, R. G., Fleming, R. F., Simons, D. S., and Newbury, D. E. (1982). Neutron-Induced Reactions, and Secondary Ion Mass Spectrometry: Complementary Tools for Depth Profiling. In Microbeam Analysis, R. F. J. Heinrich, ed., San Francisco, San Francisco Press, pp. 219–221.
Downing, R. G., Lavine, J. P., Hossain, T. Z., Russell, J. B., and Zenner, G. P. (1990). The Measurement of Boron at Silicon Wafer Surfaces by Neutron Depth Profiling. Journal of Applied Physics **67**(8), 3652–3654.
Downing, R. G., Maki, J. T., and Fleming, R. F. (1986). Application of Neutron Depth Profiling to Microelectronic Materials Processing. In Microelectronics Processing: Inorganic Materials Characterization, L. A. Casper, ed., Washington, DC, American Chemical Society, pp. 163–180.
Downing, R. G., Maki, J. T., and Fleming, R. F. (1987). Analytical Applications of Neutron Depth Profiling. Journal of Radio-analytical and Nuclear Chemistry, Articles **112**(1), 33–46.
Ehrstein, J. R., Downing, R. G., Stallard, B. R., Simons, D. S., and Fleming, R. F. (1984). Comparison of Depth Profiling B-10 in Silicon Using Spreading Resistance Profiling, Secondary Ion Mass Spectrometry, and Neutron Depth Profiling. In Third Symposium on semiconductor processing, ASTM proceedings, 850, San Jose, CA, ASTM, pp. 409–425.
Fink, D. (1983). Li, B, and N in Ancient Materials. Nuclear Instruments and Methods, 218, 456–462. Fink, D. (1988). Helium Implantation, and Thermal Annealing Behaviour. Radiation Effects **106**, 231–264.
Fink, D. (1989). Surface Precipitation of Natural, and Ion-Implanted Lithium, and Boron in Metals. Materials Science and Engineering **A115**. 89–95.
Fink, D., Biersaek, J. P., Carstanjen, H. D., Jahnel, F., Müller, K., Ryssel, H., and Osei, A. (1983). Studies on the Lattiee Position of Boron in Silicon. Radiation Effeets **77**, 11–33.
Fink, D., Biersaek, J. P., Grawe, H., Riederer, J., Müller, K., and Henkelmann, R. (1980). Applications of (n,p), and (n,*α*) Reactions, and a Backscattering Technique to Fusion Reaetor Materials, Archcometry, and Nuclear Spectroscopy. Nuelcar Instruments and Methods **168**, 453–457.
Fink, D., Biersaek, J. P., Jahnel, F., and Henkelmann, R. (1981). Untersuchung von Helium, Lithium, und Bor in Metallen mit Hilfe von (n,p), and (n,*α*)-reaktionen. In Analysis of Nonmetals in Metals, Berlin, Walter de Gruyter and Co., pp. 163–171.
Fink, D., Biersack, J. P., Kranz, H., De Souza, J., Behar, M., and Zawislak, F. C. (1988). Tilted Angle Ion Implantation. Radiation Effeets **106**, 165–181.
Fink, D., Biersaek, J. P., and Liebl, H. (1983). Background in (n,p), and (n, *α*) Spectrometry. In Ion Implantation: Equipment, and Techniques, H. Ryssel, and H. Glawisehnig, eds., Berlin, Springer-Verlag, pp. 318–326.
Fink, D., Biersaek, J. P., Müller, M., Wang, L. H., Cheng, V. K., Kassing, R., Masseli, K., Weiser, M., and Kalbitzer, S. (1989). Depth Distribution of Megaeleetronvolt ^14^N Implanted into Various Solids at Elevated Fluenees. Materials Science and Engineering **B2**, 49–54.
Fink, D., Biersack, J. P., and Städele, M. (1987). Range Profiles of Helium in Solids. Radiation Effeets **104**, 1–42.
Fink, D., Biersaek, J. P., Städele, M., Tjan, K., Behar, M., Fieht-ncr, P. F. P., Olivieri, CA., De Souza, J. P., and Zawislak, F. C. (1986). Range Profiles of Ions in Double-Layer Structures. Nuclear Instruments and Methods **B15**, 71–74.
Fink, D., Biersaek, J. P., Städele, M., Tjan, K., and Cheng, V. (1983). Nitrogen Depth Profiling Using the N^14^(n,p)C^14^ Reaction. Nuclear Instruments and Methods **218**, 171–175.
Fink, D., Biersaek, J. P., Städele, M., Tjan, K., Haring, R. A., and De Vries, R. A. (1984). Experiments on the Sputtering of Group VI Elements. Nuclear Instruments and Methods **B1**, 275–281.
Fink, D., Biersaek, J. P., Stumpff, C., and Schlosser, S. (1986). Background Reduction in Light Element Depth Profiling by a Coincidence Technique. Nuclear Instruments and Methods **B15**, 740–743.
Fink, D., Biersaek, J. P., Tjan, K., and Cheng, V. K. (1982). Ranges of He-3, and Li-6 in Various Solids. Nuclear Instruments and Methods **194**, 105–111.
Fink, D., and Fichtner, P. F. P. (1990). Unique Ion Beam Scattering Technique on Depth Profile Determination. Radiation Effeets, and Defeets in Solids **114**, 337–341.
Fink, D., Müller, M., Stettner, U., Behar, M., Fiehtner, P. F. P., Zawislak, F. C., and Koul, S. (1988). Non-Regular Depth Profiles of Light Ions Implanted into Organic Polymer Films. Nuelear Instruments and Methods **B32**, 150–154.
Fink, D., Müller, M., Wang, L., Siegel, J., Vredenberg, A., Mar-tan, J., and Fahrner, W. (1990). Energy, Fluence, and Temperature Dependence of MeV Nitrogen Implantation Profiles in Steel. Radiation Effects and Defects in Solids **115**, 121–134.
Fink, D., and Riederer, J. (1981). Studies of Li, B, and N in Aneient Oriental Pottery, and Modem Ceramic Materials by Means of (n,p), and (n, *α*) Spectrometry. Nuclear Instruments and Methods **191**, 408–413.
Fink, D., Tjan, K., Biersaek, J. P., Wang, L., and Yunru, M. (1989). Lithium Implantation Profiles in Metals, and Semiconductors. Radiation Effeets and Defeets in Solids **108**, 27–44.
Fink, D„ Tjan, K., and Wang, L. (1990), On the Thermal Mobility of Lithium in Metals, and Semiconductors. Radiation Effeets and Defeets in Solids **114**, 21–50.
Fink, D., and Wang, L. (1990). On the Thermal Annealing Behavior of Boron in Solids. Radiation Effects and Defeets in Solids **114**, 343–371.
Fink, D., Wang, L., Biersaek, J. P., and Jahnel, F. (1990). 30 keV to 2 MeV Boron Implantation Profiles in Solids. Radiation Effeets and Defeets in Solids **115**, 93–112.
Geissel, H., Lennard, W. N., Alexander, T. K., Ball, G. C., Forster, J. S., Lone, M. A., Milani, L., and Phillips, D. (1984). Influence of 1.3 MeV He-4 Post-Bombardment of the Depth Profiles of 35 keV He-3 Ions Implanted in Nb, and Au. Nuclear Instruments and Methods **B2**, 770–773.
Grasserbauer, M. (1988). Critical Evaluation of Calibration Procedures for Distributions Analysis of Dopant Elements in Silicon, and Gallium Arsenides. International Union of Pure and Applied Chemistry **60**(3), 437–444.
Guimarāes, R. B., Amarai, L., Behar, M., Fiehtner, P. F. P., Zawislak, F. G., and Fink, D. (1988). Implanted Boron Depth Profiles in the AZ111 Photoresist. Journal of Applied Physics **63**(6), 2083–2085.
Halsey, W. G. (1976). Measured Range Profile of Helium-3 in Niobium Using the ^3^He(n,p)^3^H Reaction. Masters of Seienee, University of Michigan-Ann Arbor.
Halsey, W. G. (1980). Concentration Dependent Thermal Release of Helium-3 Implantation in Molybdenum. Ph. D. thesis, University of Michigan-Ann Arbor.
Jahnel, F., Biersaek, J., Crowder, B. L., d'Heurle, F. M., Fink, D., Isaae, R. D., Lucchese, C. J., and Petrersson, C. S. (1982). The Behavior of Boron (Also Arsenie) in Bilayers of Polyerystalline Silieon, and Tungsten Disilicide. Journal of Applied Physics **53**(11), 7372–7378.
Jahnel, F., Ryssel, H., Prinke, G., Hoffmann, K., Müller, K., Biersaek, J., and Henkelmann, R. (1981). Description of Arsenie, and Boron Profiles Implanted in SiO_2_, Si_3_N_4_, and Si Using Pearson Distributions with Four Moments. Nuclear Instruments and Methods 182–183, 223–229.
Jamieson, D. N., Bowman, R. C., Jr., Adams, P. M., Knudsen, J. F., and Downing, R. G. (1988). Study of Boron Implantation in CdTc. In Fundamentals of Beam-Solid Interactions, and Transient Thermal Processing **100**, M. J. Aziz, L. E. Rehn, and B. Stritzker, eds., Pittsburgh, PA, Materials Research Society, pp. 299–304.
Khalil, N. S. (1989) Design, Installation, and Implementation of a Neutron Depth Profiling Facility at the Texas A&M Nuclear Science Center. M. S., Texas A&M University.
Knudsen, J. F., Downing, R. G., and Simons, D. S. (1987). NDP, SIMS, and Modeling of Boron Implantation Profiles in Silicon. In American Nuclear Society—Material Characterization Using Neutron Depth Profiling **55**, I. O. Macke, ed., Los Angeles, CA, American Nuclear Society, pp. 210–211.
Kotas, P., Obrusnik, J., Kvitck, J., and Hnatowicz, V. (1976). Study of Diffusion of Impurities in Semiconductor Silicon by Activation Analysis, and Nuclear Reaction Methods. Journal of Radioanalytical Chemistry **30**, 475–488.
Kristiakova, K., Kristiak, J., Kvitek, J., and Cervená, J. (1982). The Surface Boron Concentration of NixFe80-xB20 Samples. Nuclear Instruments and Methods **199**, 371.
Kvitek, J., Hnatowicz, V., and Kotas, P. (1976). Determination of Boron Concentration profiles in Silicon from B-10(n, *α*)Li-7 Reaction Product Spectra. Radiochemical and Radioanalytical Letters **24**, 205–213.
Lamaze, G. P., Downing, R. G., Langland, J. K., and Hwang, S. T. (1992). The New Cold Neutron Depth Profiling Instrument at NIST. J. Radioanal. Nucl. Chem. **160**, 315–325.
Lee, M. C., Verghese, K., and Gardner, R. P. (1988). A Model for the Detector Response Function in Neutron Depth Profiling. Nuclear Instruments and Methods **B31**, 567–575.
Lennard, W. N., Geissel, H., Alexander, T. K., Hill, R., Jackson, D. P., Lone, M. A., and Phillips, D. (1985). Depth Profiles of 35 keV ^3^He Ions in Metals. Nuclear Instruments and Methods **B10–11**, 592–595.
Lindhard, J., Scharff, M., and Schiott, H. E. (1963). Range Concepts, and Heavy Ion Ranges. Matematisk-Fysiske Meddelelser udgivet af Det Kongelige Danske Videnskabernes Selskab **33**(14), 1–42.
Losee, D. L., Hossain, T. Z., Lavine, J. P., and Downing, R. G. (1987). Neutron Depth Profiles of Ion-Implanted Boron in Polymeric Films. In American Nuclear Society—Material Characterization Using Neutron Depth Profiling **55**, Los Angeles, CA, American Nuclear Society, pp. 209–210.
Maki, J. T. (1987). Migration, and Release of Helium-3 Implanted in Single Crystal Nickel. Ph. D. thesis, University of Michigan-Ann Arbor.
Maki, J. T., Downing, R. G., and Fleming, R. F. (1985). Nitrogen Concentration Distributions by Neutron Depth Profiling. NBS Technical Note 1207, 114–118.
Maki, J. T., Fleming, R. F., and Vincent, D. H. (1985). Deconvolution of Neutron Depth Profiling Spectra. Nuclear Instruments and Methods **B17**, 147–155.
Maki, J. T., Vincent, D. H., and Fleming, R. F. (1987). Migration, and Release of ^3^He Implanted in Single-Crystal Nickel. In American Nuclear Society—Material Characterization Using Neutron Depth Profiling **55**, I. O. Macke, ed., Los Angeles, CA, American Nuclear Society, pp. 214.
Matsumura, H., Maeda, M., and Furukawa, S. (1983). Study on Impurity Diffusion in Glow-Discharged Amorphous Silicon. Japanese Journal of Applied Physics **22**(5), 771–774.
Matsumura, H., Sakai, K., Maeda, M., Furukawa, S., and Horiuchi, K. (1983). Measurement of Boron Diffusivity in Hydrogenated Amorphous Silicon by Using Nuclear Reaction B^10^(n.*α*)Li^7^. Journal of Applied Physics **54**(6), 3106–3110.
Mezey, G., Szokefalvi-Nagy, Z., and Badinka, C. S. (1973). Measurement of the Boron Distribution in B-10 Implanted Silicon by the (n,*α*) Nuclear Reaction. Thin Solid Films **19**, 173–175.
Müller, K., Henkelmann, R., Bierseck, J. P., and Mertens, P. (1977). Determination of Low Dose Concentration Profiles in Solids by Means of (n,p), and (n,*α*) Reactions. Journal of Radioanalytical Chemistry **38**, 9–17.
Müller, K., Henkelmann, R., and Boroffka, H. (1975). The Determination of Low Dose Boron Implanted Concentration Profiles in Silicon by the (n,*α*) Reaction. Nuclear Instruments and Methods 129, 557–559.
Müller, K., Henkelmann, R., Jahnel, F., Ryssel, H., Haberger, K., Fink, D., and Biersack, J. (1980). The Application of (n,*α*) Method for Boron Depth Profiling, and Channel-Blocking Measurements in Semiconductor Materials. Nuclear Instruments and Methods **170**, 151–155.
Myers, D. J. (1979) Range Profiles of Helium in Copper After Thermal Anneals. Masters, University of Michigan-Ann Arbor.
Myers, D. J., Halsey, W. G., King, J. S., and Vincent, D. H. (1980). He-3 Release from Copper. Radiation Effects **51**, 251–252.
Nagy, A. Z., Bogáncs, J., Gyulai, J., Csoke, A., Nazarov, V., Seres, Z., Szabó, A., and Yazvitsky, Y. (1977). Determination of Boron Range Distribution in Ion-Implanted Silicon by the B^10^(n,*α*)Li^7^ Reaction. Journal of Radioanalytical Chemistry **38**, 19–27.
Nagy, A. Z., Vasvari, B., Duwez, P., Bakos, L., Bogáncs, J., and Nazarov, V. M. (1980). Variation of Boron Concentration in Metallic Glass Ribbons. Physics Status Solidi (a), **61**, 689–692.
Parikh, N. R., Chu, W. K., Wehring, B. W., and Miller, G. D. (1987). Boron-10 Distribution in Silicon, TiSi_2_, and SiO_2_ Using Neutron Depth Profiling. In American Nuclear Society—Material Characterization Using Neutron Depth Profiling **55**, I. O. Macke, ed., Los Angeles, CA, American Nuclear Society, pp. 211–212.
Parikh, N. R., Frey, E. C., Hofsäs, H. C., Swanson, M. L., Downing, R. G., Hossain, T. Z., and Chu, W. K. (1990). Neutron Depth Profiling by Coincidence Spectrometry. Nuclear Instruments and Methods **B45**, 70–74.
Pelikan, L., Rybka, V., Krcjci, P., Hnatowicz, V., and Kvitek, J. (1982). Study of Boron Implantation in Ag-Si Layer Structures. Physics Status Solidi **72**, 369–373.
Riley, J. E., Jr. (1987). The Effects of Lithium Isotopie Anomalies on Lithium Niobate. Ferroelectrics **75**, 59–62.
Riley, J. E., Jr., and Downing, R. G. (1987). Quantitative Determination of Boron in Semiconductors Using Neutron Depth Profiling. In American Nuclear Society—Material Characterization Using Neutron Depth Profiling **55**, I. O. Macke, ed., Los Angeles, CA, American Nuclear Society, pp. 207.
Riley, J. E., Jr., Downing, R. G., and Fleming, R. F. (1987). Neutron Depth Profiling of Lithium in Lithium Niobate. In American Nuclear Society—Material Characterization Using Neutron Depth Profiling 55, Los Angeles, CA, American Nuclear Society, pp. 214–215.
Riley, J. E., Jr., Mitchell, J. W., Downing, R. G., Fleming, R. F., Lindstrom, R. M., and Vincent, D. M. (1984). Material Analysis Using Thermal Neutron Reactions: Applications. Materials Science Forum **2**, 123–132.
Ryssel, H., Habcrger, K., Hoffmann, K., Prinke, G., Dumckc, R., and Sachs, A. (1980). Simulation of Doping Processes. IEEE Transactions on Electron Devices IEEE Transactions on Electron Devices **ED27**(8), 1484–1492.
Ryssel, H., Kranz, H., Müller, K., Henkelmann, R. A., and Biersack, J. (1977). Comparison of Range, and Range Straggling of Implanted B-10, and B-11 in Silicon. Applied Physics Letters **30**(8), 399–401.
Ryssel, H., Müller, K., Biersack, J. P., Kruger, W., Lang, G., and Jahuel, F. (1980). Range, and Range Straggling of Ion-Implanted Boron in Cd0.2 Hg0.8 Te. Physics Status Solidi **57**, 619–624.
Ryssel, H., Müller, K., Haberger, K., Henkelmann, R., and Jahuel, F. (1980). High Concentration Effects on Ion Implanted Boron in Silicon. Applied Physics **22**, 35–38.
Ryssel, H., Prinke, G., Haberger, K., Hoffmann, K., Müller, K., and Henkelmann, R. (1981). Range Parameters of Boron Implanted into Silicon. Applied Physics **24**, 39–43.
Thwaites, D. I. (1983). Bragg's Rule of Stopping Power Additivity: A Compilation, and Summary of Results. Radiation Research **95**, 495–518.
Tjan, K., Fink, D., Bicrsack, J. P., and Städele, M. (1986). Implantation Profiles of Li in Metals. Nuclear Instruments and Methods **B15**, 54–57.
Ünlü, K. (1989). Helium-3 in Nickel Base Amorphous Metals: Surface Features, Subsurface Microstructure, Migration, and Release Upon Annealing. Ph. D. thesis, University of Michigan-Ann Arbor.
Ünlü, K., and Vincent, D. H. (1990). Range Profiles, and Thermal Release of ^3^He Implanted into Various Nickel-Based Amorphous Alloys. Nuclear Instruments and Methods **A299**, 606–609.
Usmanova, M. M., Zverev, B. P., Simakhin, Y. F., Idrisov, K., and Zhumaev, N. (1984). Analysis of Distribution of Impurities by Using Neutron Beams. Yad. Fiz. Mctody Kor. Pol. Mat. Met. 5–13.
Vandervorst, W., Shepherd, F. R„ and Downing, R. G. (1985). High Resolution SIMS, and Neutron Depth Profiling of Boron Through Oxide-Silicon Interfaces. Journal of Vacuum Science and Technology **A3**(3), 1318–1321.
Vodopyanov, L. K., and Kozyrev, S. P. (1982). Ion Implantation of B^+^ in n-Hg0.8 Cd0.2 Te. Physics Status Solidi **72**, K133–K136.
Wilson, S. R., Gregory, R. B., Paulson, W. M„ Krause, S. J., Gressett, J. D., Hamdi, A. H., McDaniel, F. D., and Downing, R. G. (1985). Properties of Ion-Implanted Polycrystalline Si Layers Subject to Rapid Thermal Annealing. Journal of the Electrochemical Society **132**(4), 922–929.
Zeitzoff, P. M., Hossain, T. Z., Boisvert, D. M., and Downing, R. G. (1990). Measurement, and Control of the Boron, and Phosphorus Concentration in LPCVD Borophosphosilicate Glass. Journal of the Electrochemical Society **137**(12), 3917–3922.
Ziegler, J. F., Cole, G. W., and Baglin, J. E. E. (1972). Techniques for Determining Concentration Profiles of Boron Impurities in Substrates. Journal of Applied Physics **43**(9).
Ziegler, J. F., Crowder, B. L., Cole, G. W., Baglin, J. E. E., and Masters, B. J. (1972). Boron Atom Distributions in Ion-Implanted Silicon by the (n,^4^He) Nuclear Reaction. Applied Physics Letters **21**, 16–17.

## References

[1] Downing RG, Fleming RF, Langland JK, Vincent DH (1983). Nucl Instr Mcth.

[2] Ziegler JF, Colc GW, Baglin JEE (1972). J Appl Phys.

[3] Fink D, Biersack JP, Liebl H, Ryssel H, Glawischnig H (1983). Ion Implantation: Equipment and Techniques.

[4] Myers DJ (1979). Range Profiles of Helium in Copper After Thermal Anneals.

[5] Myers DJ, Halsey WG, King JS, Vincent DH (1980). Radia Eff.

[6] Halsey WG (1980). Concentration Dependent Thermal Release of Hclium-3 Implantation in Molybdenum.

[7] Khalil NS (1989). Design, Installation, and Implementation of a Neutron Depth Profiling Facility at the Texas A&M Nuclear Science Center.

[b8-jresv98n1p109_a1b] 8K. Ünlü, Private Communication (1990).

[9] Parikh NR, Chu WK, Wehring BW, Miller GD (1987). Boron-10 Distribution in Silicon, TiSi_2_, and SiO_2_ Using Neutron Depth Profiling 1.

[10] Ziegler JF (1977).

[11] Janni JF (1982). Atom Nucl Data Tabl.

[12] Thwaites DI (1983). Radia Research.

[13] Nieman HF, Tennant DC, Dolling G (1980). Rev Sci Instr.

[14] Biersaek JP, Fink D, Lauch J, Henkelmann R, Müller K (1981). Nucl Instr Meth.

[15] Fink D (1983). Radia Eff.

[16] Bogáncs J (1979). Radiochem Radioanal Lett.

[17] Crowder BL, Ziegler JF, Cole GW, Crowder BL (1973). Ion Implantation in Semiconductors, and Other Materials.

[18] Matsumura H, Sakai K, Maeda M, Furukawa S, Horiuchi KJ (1983). Appl Phys.

[19] Müller K, Henkelmann R, Bierseck JP, Mcrtcns PJ (1977). Radioanal Chcm.

[20] Nagy AZ (1977). J Radioanal Chem.

[21] Lamaze GP, Downing RG, Langland JK, Hwang ST (1992). J Radioanal Nucl Chem.

[22] Deruyttcr AJ, Pelfer PJ (1967). Nucl Energy.

[23] Biersack JP, Fink D, Henkelmann R, Müller K (1978). Nucl Instr Mcth.

[24] Maki JT, Fleming RF, Vincent DH (1985). Nucl Instr Meth.

[25] Ccrvoná J (1981). Nucl Instr Meth.

[26] Bogáncs J (1979). Joint Instit Nucl Res.

[27] Kvitek J, Hnatowicz V, Kotas P (1976). Radiochem Radioanal Lett.

[28] Ryssel H (1980). IEEE Trans, on Elect Dev.

[29] Nagy AZ (1980). Physics Status Solidi (a).

[30] Ccrvená J (1981). Tesla Elect.

[31] Parikh NR (1990). Nucl Instr Meth.

[32] Fink D (1986). Nucl Instr Meth.

[33] Mildner DFR (1990). Nucl Instr Meth.

[34] Ehrstcin JR, Downing RG, Stallard BR, Simons DS, Fleming RF (1984). Comparison of Depth Profiling B-10 in Silicon Using Spreading Resistance Profiling, Secondary Ion Mass Spectrometry, and Neutron Depth Profiling 1.

[35] Fink D (1988). Radia Eff.

[36] Cox JN, Hsu R, McGregor PJ, Downing RG (1987). NDP and FTIR Studies of Borophosphosilicate CVD Thin-Film Glasses 1.

[37] Jamieson DN, Bowman RC, Adams PM, Knudsen JF, Downing RG (1988). Study of Boron Implantation in CdTe 1.

[38] Crowder BL, Ziegler JF, Morehead FF, Cole GW, Crowder BL (1973). Ion Implantation in Semiconductors, and Other Materials.

[39] Ziegler JF, Crowder BL, Cole GW, Baglin JEE, Masters BJ (1972). J Appl Phys.

[40] Müller K (1980). Nucl Instr Meth.

[41] Ryssel H (1981). Appl Phys.

[42] Geissel H (1984). Nucl Instr Meth.

[43] Biersack JP, Fink D, Namba S (1975). Ion Implantation in Semiconductors.

[44] Downing RG, Lavine JP, Hossain TZ, Russell JB, Zenner GP (1990). J Appl Phys.

[45] Ryssel H (1980). Physiea Status Solidi.

[46] Vodopyanov LK, Kozyrev SP (1982). Physics Status Solidi.

[47] Bowman RC, Robertson RE, Knudsen JF, Downing RG (1986). Studies of Boron Implantations through Photochemically Deposited SiO_2_ Films on Hg1-xCdxTe 1.

[48] Jahnel F (1982). J Appl Phys.

[49] Pelikan L, Rybka V, Krejci P, Hnatowicz V, Kvitek J (1982). Physiea Status Solidi.

[50] Ryssel H, Müller K, Haberger K, Henkelmann R, Jahnel F (1980). Appl Phys.

[51] Matsumura H, Maeda M, Furukawa S (1983). Japan J Appl Phys.

[52] Biersack JP, Fink D, Datz S, Appleton BR, Moak CD (1975). Atomic Collisions in Solids.

[53] Biersack JP, Fink D (1973). Nucl Instr Mcth.

[54] Riley JE (1984). Mater Sci Forum.

[55] Downing RG, Maki JT, Fleming RF, Casper LA (1986). Microelectronics Processing: Inorganic Materials Characterization.

